# HIV incidence estimate combining HIV/AIDS surveillance, testing history information and HIV test to identify recent infections in Lazio, Italy

**DOI:** 10.1186/1471-2334-12-65

**Published:** 2012-03-20

**Authors:** Alessia Mammone, Patrizio Pezzotti, Claudio Angeletti, Nicoletta Orchi, Angela Carboni, Assunta Navarra, Maria R Sciarrone, Catia Sias, Vincenzo Puro, Gabriella Guasticchi, Giuseppe Ippolito, Piero Borgia, Enrico Girardi

**Affiliations:** 1 Istituto Nazionale per le Malattie Infettive "Lazzaro Spallanzani", Rome, Italy; 2 Laziosanità--Agenzia di Sanità Pubblica, Rome, Italy

**Keywords:** HIV incidence, Test for recent infection, Testing history, Avidity index

## Abstract

**Background:**

The application of serological methods in HIV/AIDS routine surveillance systems to identify persons with recently acquired HIV infection has been proposed as a tool which may provide an accurate description of the current transmission patterns of HIV. Using the information about recent infection it is possible to estimate HIV incidence, according to the model proposed by Karon et al. in 2008, that accounts for the effect of testing practices on the number of persons detected as recently infected.

**Methods:**

We used data from HIV/AIDS surveillance in the period 2004-2008 to identify newly diagnosed persons. These were classified with recent/non-recent infection on the basis of an avidity index result, or laboratory evidence of recently acquired infection (i.e., previous documented negative HIV test within 6 months; or presence of HIV RNA or p24 antigen with simultaneous negative/indeterminate HIV antibody test). Multiple imputation was used to impute missing information. The incidence estimate was obtained as the number of persons detected as recently infected divided by the estimated probability of detection. Estimates were stratified by calendar year, transmission category, gender and nationality.

**Results:**

During the period considered 3,633 new HIV diagnoses were reported to the regional surveillance system. Applying the model, we estimated that in 2004-2008 there were 5,465 new infections (95%CI: 4,538-6,461); stratifying by transmission category, the estimated number of infections was 2,599 among heterosexual contacts, 2,208 among men-who-have-sex-with-men, and 763 among injecting-drug-users. In 2008 there were 952 (625-1,229) new HIV infections (incidence of 19.9 per 100,000 person-years). In 2008, for men-who-have-sex-with-men (691 per 100,000 person-years) and injecting drug users (577 per 100,000 person-years) the incidence remained comparatively high with respect to the general population, although a decreasing pattern during 2004-2008 was observed for injecting-drug-users.

**Conclusions:**

These estimates suggest that the transmission of HIV infection in Lazio remains frequent and men-who-have-sex-with men and injecting-drug-users are still greatly affected although the majority of new infections occurs among heterosexual individuals.

## Background

Estimating HIV incidence is essential for monitoring the evolution of the epidemic and evaluating the effectiveness of prevention efforts. However, providing accurate estimates of HIV incidence is a complex task. Measuring the seroconversion rate in cohort studies or repeated serosurveys is expensive and at best reveal incidence in high-risk groups [1] while routine surveillance systems record new HIV diagnoses and not new infections [2].

The application of new laboratory techniques in HIV/AIDS routine surveillance systems to identify persons with recently acquired HIV infection has been proposed as a tool which may provide an accurate description of the current transmission patterns of HIV. These techniques are based on serological tests, usually defined as tests for recent infection (TRI), which use different algorithms to discriminate recent infections from long-standing ones using a single serum sample [3-5]. Their development was based on the dynamics of the humoral immune response during the post-seroconversion phase of HIV infection, and relies on the modifications of early HIV-1 antibodies over time during the early phase of the infection [6-8]. TRI are likely able to identify HIV infections that occurred within 5-7 months before the test with an overall median sensitivity of 88.8% (range 42.3-100%) and a median specificity of 86.8% (range 49.5-100%) [9], and some developed countries have implemented surveillance with TRI [10-16] either at a regional or national level. However, identifying recent infections does not directly provide an estimate of the incidence of HIV infection because persons who have recently been infected can delay the testing and are not necessarily tested in the first months after infection. Karon et al. proposed a statistical model combining HIV/AIDS surveillance data with TRI and testing history that provided estimates of incidence of HIV infection in the US [16,17]. Another study following a similar approach provided incidence estimates for France [18].

In Lazio, a region located in central Italy including the metropolitan area of Rome (almost six million inhabitants), a combined surveillance of HIV and AIDS cases has been active since 1985, [19] and since 2004, a TRI is performed in a substantial percentage of new HIV diagnoses through a multicentre study [20]. The test used to detect recent infections is based on measuring the Avidity Index (AI) of the HIV-1 specific antibodies which show a low avidity for the antigen in the early phase of the infection [21-23]. It can be performed by an automated enzyme immunoassay for antibodies to HIV, it is not expensive and has been shown to identify recent HIV infections with good accuracy [22-25].

The objective of this study was to provide an estimate of the incidence of HIV infection in this Italian region by using data provided by the routine surveillance system and TRIs, using the method proposed by Karon et al. [17].

## Methods

### The regional surveillance system of HIV and AIDS diagnosis of the Lazio region

Although a national HIV surveillance system was not implemented in Italy until 2009, in Lazio region, a mandatory Regional Surveillance System of HIV infections - based on the anonymous notification of every HIV diagnosis by public and private laboratories and blood banks- has been established since 1985 [19]. HIV diagnosis is defined as a positive result on two consecutive assays for HIV antibodies performed with commercially available immunoenzymatic tests and confirmed by a positive Western blot.

For each individual diagnosed with HIV, laboratories fill a form with some personal identifying data [i.e., gender, date (i.e., day, month and year) and municipality of birth (country if born outside Italy) but not surname and name]. Then the form is sent to the regional AIDS units where the test result is given to the patient, and other information (i.e., risk factors, date of last documented negative test when available) are collected during the post-test counselling. All information are finally collected and analysed at regional level.

In order to identify multiple tests of the same subject, a linkage procedure, based on gender, date and municipality of birth is routinely performed on the HIV diagnosis reports. This generates a file of newly diagnosed HIV infection which is periodically updated. It is of note that this procedure can identify different individuals with the same gender, date and municipality of birth. However, when we simulated the specificity of this combination of information, using the National AIDS registry (see below for description), that collects all the personal identifying data for more than 60,000 cases, we found around 1 duplicate every 2,000 AIDS cases (i.e., 0.5 per 1,000). To ensure confidentiality, this file is protected by safety procedures and can only be accessed for the purpose of surveillance.

Since 1985, following the indications of the Italian AIDS registry, in Lazio Region AIDS diagnoses are reported mandatorily. The case definition refers to the European definition. Complete identifying personal data are given in the form used and are routinely linked to the HIV diagnoses using a linkage procedure based on gender, date and municipality of birth. The combination of the two surveillance systems is called the Regional Surveillance System of HIV and AIDS (RSS).

In this study we selected all newly diagnosed HIV infections identified in the period from 2004-2008.

### The SENDIH study

The SENDIH (Studio Epidemiologico Nuove Diagnosi Infezione HIV-1) is a multicentre study which started in 2004. Characteristics and methods of the study have been previously described [20]. Briefly, the study collects the following information on newly diagnosed adults with HIV infection from 13 regional counselling and testing sites of the Lazio region: demographic data (age, gender, nationality, transmission category), date of last HIV documented negative test, clinical and laboratory data at diagnosis (including the presence of AIDS defining conditions, diagnosis of primary HIV infection, HIVRNA measurement, and CD4 cell count), and, after the individual has provided written informed consent, behavioural data. The study was approved by the ethics committee at the L. Spallanzani National Institute of Infectious Diseases, and all enrolled individuals provided written informed consent.

### Linkage procedure between RSS and SENDIH

Given that neither RSS nor SENDIH collect complete identifying personal data, each centre participating in the SENDIH study was asked to send a file in which there was a record for each diagnosis containing both of the anonymous codes used for the RSS and the SENDIH. The data were then combined using these files.

### Definitions of recent and non recent infection

Only individuals enrolled in SENDIH study could have been classified as having recent or long-standing infection. Recent infection identification was based on three different criteria: 1) if an individual had a documented negative HIV antibody test performed within 6 months before HIV diagnosis; 2) if he/she had laboratory evidence of HIV seroconversion at the time of diagnosis [i.e., presence of HIV RNA or HIV p24 antigen with simultaneous negative/indeterminate HIV antibody testing (HIV-1/2 ELISA and Western Blot)]; 3) an AI < 0.80. To save laboratory resources, when one of the first two criteria is met, the AI is not performed because the individual is considered as having AI < 0.80.

An AI of HIV antibodies test was performed only if a serum sample was available within 2 months after the initial diagnosis, if the patient had a CD4 count ≥ 20 cells/μL and was clinical-AIDS-free. Moreover, since early treatment was found to affect the evolution of HIV antibody avidity [23], in newly diagnosed individuals who started anti-retroviral therapy, AI was not performed.

The AI of antibodies is calculated by an automated anti-HIV enzyme immunoassay (EIA), the AxSYM HIV 1/2gO (Abbott Diagnostics Division, Delkenheim, Germany), according to a procedure already described by Selleri [23]. An AI lower than 0.80 was selected to define a recent infection because identified as threshold with the highest accuracy (area under the receiver operating curve: 0.958) corresponding to a sensitivity of 93.0% and a specificity of 98.5% [26]; this threshold was found to be associated with a mean window period of 202 days (standard error: 18.4 days) [27].

Conversely, patients were classified as having non-recent infection if they had an AI ≥ 0.80.

### Incidence estimate of HIV infection

To estimate the regional incidence of HIV infection, we used the model proposed by Karon et al. [17] that accounts for the effect of testing practices on the number of persons detected as being recently infected.

According to this method, incidence is estimated as the number of persons detected as being recently infected divided by the estimated probability of being detected as recently infected during the period of interest. The incidence estimator is:

$$I=r/(p_{1}{\;}^{*}p_{2}{\;}^{*}p_{3}),$$

where r is the number of recent infections detected, p_1 _represents the estimated probability of having a HIV test within one year after infection (in brief it represents the population testing behaviour), which is different for individuals having a previous negative HIV test (defined as repeat testers) and for individuals at their first HIV test (new testers); p_2 _is the percentage of persons with a newly reported HIV infection that had a TRI result; and p_3 _is the probability of being classified as recently infected, given a sample obtained at most one year after infection.

Incidence of HIV infection was firstly estimated using original data, assuming that the testing history data and the AI results are missing completely at random. However, in a logistic regression model, significant predictors (p < 0.001) of missing testing history information were transmission category (three categories: MSM, IDU and heterosexual contacts) and type of diagnosis (four categories: AIDS diagnosis, (at HIV or within six months from HIV diagnosis), recent infection, non recent infection and HIV diagnosis AIDS-free with no criterion available for defining recent infection), while significant predictors (p < 0.001) of missing AI results were transmission category, nationality (two categories: Italians and non-Italians), and history of previous HIV testing (two categories: Yes or No).

We then assumed that data on testing history and the AI results were missing at random and missing data were estimated by a two stage multiple imputation procedure.

First, history of previous HIV testing based on transmission category and type of diagnosis was imputed; at the same stage, conditionally on a previous imputed negative test, time delay between the two tests was imputed using a linear regression model based on transmission category and type of diagnosis as described above and 5 datasets were generated. Second, the AI results based on transmission category, nationality and history of previous HIV testing were imputed and 4 datasets were imputed from those obtained at the first stage (20 datasets in total).

For the original data and for each generated dataset with no missing information, for repeat testers, p_1_^RT ^was estimated as the mean inverse inter-test time for repeat testers having a known last negative test date. For new testers, p_1_^NT ^was estimated considering the proportion of HIV infections diagnosed at the AIDS stage and the distribution of the AIDS incubation periods, corresponding to a median incubation time of 8 years between infection and AIDS [28]. We used the US Centers for Disease Control and Prevention AIDS case definition [29] which is based on the presence of an AIDS defining illness or a CD4 count < 200 cells/μL.

When estimating the incidence of HIV infection with original data, p_2 _was estimated separately for repeat and new testers; since p_2 _represents the probability that a person diagnosed with HIV had a TRI that could result in being classified as recent, p_2 _is estimated not considering persons with an initial diagnosis of AIDS or who develop AIDS within 6 months of their HIV diagnosis. When using the generated dataset with no missing information p_2 _is one.

For p_3_, a mean window period for an AI < 0.80 of 202 days was considered [27]. We assumed that also patients not tested with AI but identified as recently acquired infections with the other criteria have an AI < 0.80.

For each dataset, the total incidence estimate was then the sum of the estimated incidence for new testers,

$${\text{I}}^{\text{NT}}={\text{r}}^{\text{NT}}/\left({{\text{p}}_{1}}^{\text{NT}}\;{{\text{p}}_{2}}^{\text{NT}}{\text{p}}_{3}\right)$$

and the estimated incidence for repeat testers,

$${\text{I}}^{\text{RT}}={\text{r}}^{\text{RT}}/\left({{\text{p}}_{1}}^{\text{RT}}\;{{\text{p}}_{2}}^{\text{RT}}{\text{p}}_{3}\right).$$

Variances and 95% confidence intervals reported were calculated using the method recently proposed by Carnegie [30].

For the imputed datasets, the estimates reported are the mean of the 20 values obtained, while the lower and the upper bound of the 95%CI were assumed to be respectively the minimum of the lower bound and the maximum of the upper bound, among the 20 estimates.

Estimates were stratified by calendar year, gender, transmission category and nationality (i.e., Italian, non-Italian).

To estimate incidence rates (overall or stratified by calendar year or gender), we used annual estimates of the population aged 15 years old or more residing in the region of Lazio from the National Bureau of Census http://www.demo.istat.it. The number of injecting drug users were obtained using the prevalence of heroin users in Italy from 2004-2008 estimated by the European Monitoring Centre for Drugs and Drug Addiction, corrected by the proportion of injecting subjects [31]. The estimated size of the non-national population living in Lazio, including documented and non-documented immigrants, was obtained from the Dossier Statistico Caritas/Migrantes [32]. The number of men who have sex with men was calculated as 3.1% of males aged 18-70, based on the results of a survey on sexual behaviours recently conducted in Italy [33].

Analyses were done with R software version 2.12 [34]. In particular package mi [35] was used for multiple imputation and package hivi [30] was used to compute 95% confidence intervals of estimates.

## Results

During the period 2004-2008, 3,633 new HIV diagnoses were reported to the RSS within the end of 2010 (Table 1). Among them, 2,170 (60%) were diagnosed in SENDIH sites. Individuals diagnosed in SENDIH were more frequently men (79% vs. 70% in the non-SENDIH), MSM (53% vs. 26%) and less frequently heterosexuals (38% vs. 63%); no difference was observed by age and for the percentage of IDU and foreigners. Of the 3,633 HIV diagnoses, 886 (24.4%) were classified as having AIDS according to the CDC definition (of whom 69.4% had a CD4 count < 200 cells/μL and no AIDS-defining illness) and a further 512 (14%) were classified as long-standing infections based on AI results. Two hundred ninety (8%) individuals were classified as recent infections: in 52% of the cases classification was based on AI results, 30% had a documented negative HIV test in the previous 6 months, while the remaining 18% had laboratory evidence of HIV seroconversion. One thousand-nine-hundred-forty-five individuals (53.5% out of the total, 34% out of those diagnosed in SENDIH sites) were AIDS-free at the time of diagnosis of HIV but no criterion to establish a recent infection was available. Information about the history of previous HIV testing was available for 44.2% of all cases: the proportion of recent infection was 32.2% and 8.6% among classifiable patients with and without a previous HIV test, respectively.

**Table 1:** New HIV diagnoses by type of diagnosis and information on a previous negative HIV test; Lazio, Italy, 2004-2008

	Ever Tested HIV Negative?			
	
	Yes	No	Unknown	Total
AIDS* within 6 months of HIV diagnosis	19	65	100	184
AIDS* diagnosis at HIV diagnosis	102	324	276	702
**HIV diagnosis, non-recent infection**	278	194	40	512
**HIV diagnosis, recent infection**	190	55	45	290
**HIV diagnosis, no test for recent infection**	201	177	1,567	1,945

**Total**	**790**	**815**	**2,028**	**3,633**

*Diagnoses at the AIDS stage were considered as non-recent regardless of avidity index results. AIDS definition used: clinical AIDS or CD4 count < 200 cells/μL

After the multiple imputation procedure, overall, during the period 2004-2008 we estimated 5,465 new infections in the region of Lazio (95%CI: 4,538-6,461) with an average of 1,099 (23.8 per 100,000) estimated infections per year (Table 2).

**Table 2:** Parameter estimates, incidence estimates and 95% confidence intervals (based on both the original and imputed data), Lazio, Italy, 2004-2008

	Original data		Imputed data^†^	
	Repeat testers	New testers	Repeat testers	New testers
**r**	190	55	620	301
**p_1_**	0.522	0.136	0.398	0.205
**p_2_**	0.696	0.585	1	1
**Incidence estimate**	946	1,250	2,818	2,647
**(95% CI)**	(857-1,035)	(966-1,534)	(2,305-3,426)	(1,739-3,593)
**Diagnoses**	790	815	1,646	1,987
**Total incidence estimate (95% CI)**	**4,968** (4,356-5,441)		**5,465** (4,538-6,461)	
Estimated incidence rate*	**21.1**		**23.8**	

* per 100,000 person-years; estimates refer to the mean population (aged 15 or older) in the region of Lazio during the period 2004-2008.

^**†**^mean values from 20 imputations.

P_3 _= 202/365.25 = 0.553.

CI, Confidence Interval.

The number of newly diagnosed cases per year was quite stable during the study period (an average of 723 new diagnoses per year, range 682-786) as it was the number of estimated identified recent infections, except for a peak observed in 2006 (Table 3). The estimated infections per year varied greatly, ranging from a minimum of 952 (19.9 per 100,000) in 2008 to a maximum of 1,223 (26.8) in 2006 (Table 3).

**Table 3:** Estimates of HIV incidence infections per calendar year, region of Lazio, Italy, 2004-2008

Year of diagnosis	Newly diagnosed HIV infections	Identified Recent Infections N(%)*	Estimate infections	95% Confidence Interval	Estimated Incidence rate**
**2004**	760	184 (24)	1,144	843 -- 1,516	25.5
**2005**	695	185 (27)	1,190	892 -- 1,567	26.2
**2006**	786	216 (27)	1,223	925-- 1,636	26.8
**2007**	710	169 (24)	982	719 -- 1,328	20.8
**2008**	682	167 (24)	952	625-- 1,229	19.9
**Total**	**3,633**	**921**			

* mean values from 20 imputations;% refers to the total of new diagnoses of each year

** per 100,000 person-years; estimates refer to the population (aged 15 or older) in the region of Lazio on January 1^st ^of each year

When data were analyzed according to transmission category (Table 4), the higher number of new diagnoses was observed among heterosexual contacts (48.6% of the total diagnoses) followed by MSM (41.9%). The highest estimated number of identified recent infections was among MSM (440 out of a total of 921 estimated as identified during the study period, 48%) and in this group there was a lower proportion of AIDS cases. The highest number of new HIV infections was estimated to have occurred among heterosexual contacts (2,599, 47% out of the total), followed by MSM (2,208, 40%) and by IDU (763, 13%).

**Table 4:** Estimated new HIV infections, region of Lazio, Italy, 2004-2008, by transmission category (mean values from 20 imputations)

	Heterosexual contacts	MSM	IDU
**Total newly diagnosed HIV infections**	**1,765**	**1,523**	**345**

Identified recent infection (%)*	371 (21)	440 (29)	110 (32)

Non-recent infection (%)*	920 (52)	765 (50)	141 (41)

AIDS case (%)*	474 (27)	318 (21)	94 (27)

**Total incidence estimate (95% CI)**	**2,599** (1,930-3,386)	**2,208** (1,641-2,689)	**763** (476-1,059)

*% refers to the total number of diagnoses in each group

MSM, Men who have Sex with Men; IDU, Injecting Drug Users; CI, Confidence Interval

With respect to gender and place of birth (Table 5), men account for 76% of the new diagnoses and persons born in Italy for 67%, respectively; the estimated percentage of new diagnoses classified as recent infections tended to be higher in men than in women and in those born in Italy compared to foreign born individuals. The number of new infections estimated for men was approximately three times the number estimated for women, and cases estimated for Italians were slightly more than twice of those estimated for non-Italians (Table 5).

**Table 5:** Estimated new HIV infections, region of Lazio, Italy, 2004-2008, by gender and nationality (mean values from 20 imputations)

	MEN	WOMEN	ITALIANS	NON ITALIANS
**Total newly diagnosed HIV infections**	**2,727**	**906**	**2,374**	**1,259**

Identified recent infection (%)*	715 (26)	206 (23)	640 (27)	281 (22)

Non-recent infection (%)*	1,319 (49)	507 (56)	1,139 (48)	687 (55)

AIDS case (%)*	693 (25)	193 (21)	595 (25)	291 (23)

**Total incidence estimate (95% CI)**	**4,197** (3,373-4,978)	**1,280** (875-1,774)	**3,773** (2,886-4,617)	**1,703** (1,159-2,210)

*% refers to the total number of diagnoses in each group

CI, Confidence Interval

Table 6 shows the estimated incidence rates in some sub-groups (i.e., MSM, IDU, and non-Italians) by calendar year. Compared to estimates reported for the general population, on average, these rates are 30 times higher in MSM, 40 times higher for IDU and 3 times higher for non-Italians. Time trends in incidence for MSM and non-Italians were similar to the overall trend, while a constant decreasing trend was observed for IDU.

**Table 6:** Estimated new HIV infections and incidence, region of Lazio, Italy, 2004-2008, by transmission category and nationality

	Year	Estimated infections*(95% CI)	Estimated population size§	Estimated incidence rate**
**MSM**	**2004**	466 (267-714)	55,853	834
	**2005**	488 (335-676)	56,479	864
	**2006**	506 (292-714)	56,641	893
	**2007**	352 (229-504)	58,596	601
	**2008**	408 (238-561)	59,043	691
**IDU**	**2004**	216 (83-350)	15,784	1,368
	**2005**	195 (59-400)	11,752	1,659
	**2006**	158 (64-298)	12,666	1,247
	**2007**	112 (8-215)	13,286	843
	**2008**	74 (6-181)	12,818	577
**NON-ITALIANS**	**2004**	285 (143-420)	389,920	73
	**2005**	389 (172-553)	418,823	93
	**2006**	411 (211-683)	500,007	82
	**2007**	331(144-509)	480,700	69
	**2008**	292 (128-480)	450,151	65

* mean values from 20 imputations

§Population size estimates on January 1^st ^of each year; aged 18-70 years for MSM, 15-64 for IDU

**Per 100,000 person-years

MSM, Men who have Sex with Men; IDU, Injecting Drug Users; CI, Confidence Interval

## Discussion

During its first decade, the HIV epidemic in Italy was predominantly characterized by infections linked to intravenous drug use followed by those occurring among MSM. In this study we show that during the third decade of the epidemic, the majority of new HIV infections in an Italian region can be estimated to occur among heterosexual individuals. The HIV epidemic however, continues to disproportionately affect MSM and IDU. The overall estimated incident rates estimated for the period from 2004-2008 are of the same order of magnitude of those estimated for the 1990s, when the epidemic (according to the estimates based on back calculation methods) was stabilizing after an initial peak [36].

This is the first study that provides recent estimates of HIV incidence in an Italian region. The last estimates reported for the region of Lazio referred to 1992 with an estimate of around 2,000 new infections from a dynamic model [37]; in contrast, by using a back-calculation model, Bellocco et al. [38] estimated only 2500-3000 infections in Italy during 1994, corresponding to around 300-350 infections in Lazio region (based on the assumption that Lazio region has quite constantly accounted for 12% of the Italian AIDS cases each year and that this percentage holds also in the new HIV infections [39]). According to our estimates, in the period from 2004-2008 there was an average of around 1,099 infections per year. Although it is difficult to compare our results with previous estimates, the mean number of infections estimated for 2004-2008 remains surprisingly high.

There are no data available to provide a national estimate of HIV incidence by using this method. However, on the basis of our results it would be possible to provide a rough extrapolation of the expected number of new infections in the country, since HIV diagnosis and AIDS incidence in Lazio and the rest of Italy have similar temporal trends and similar characteristics in terms of gender, age and risk groups, [39] and given that there is free offer of treatment for infected persons in the whole country. In fact, considering that the region of Lazio has quite constantly accounted for 12% of the Italian AIDS cases each year, [39] and considering a mean estimated number of infections of around 1,099 cases each year in Lazio region, we could roughly estimate around 9,100 new infections each year in the entire country, which corresponds to an estimated incidence of around 15 per 100,000.

Our incidence estimates generally varied by calendar year but there was no specific trend. While the peak was estimated in 2006, stratified analyses showed similar results in each sub-group except for injecting drug users for whom there was a clear decline of the estimated recent infections in the period from 2004-2008.

The statistical model used in this paper has been previously applied in the US and in France. Incidence estimates of 19.0 per 100,000 were obtained for the US [40] and of 17 per 100,000 for France, both in 2008 [18]; these estimates are similar to those obtained in the region of Lazio in the period 2004-2008.

Estimates of incidence rates by risk group provided important information about the HIV epidemic in our region. Regarding drug injection, the estimated number of new infections related to this behaviour had a decreasing trend during the period from 2004-2008, and new infections related to this behaviour represented approximately 14% of the overall cases estimated during the study period. However, in 2008 the estimated incidence rate was around 200 per 100,000, suggesting that IDU are still at high risk of infection. Also the MSM, who accounted for almost 40% of estimated infections, had an alarmingly high incidence rate that was around or above 500 per 100,000 in each year of the study. These results are consistent with those reported in France [18], where IDU and MSM were found to be disproportionately affected by the HIV epidemic, underscoring the urgent need to reinforce the prevention interventions that are targeted to these groups.

In this study we did not attempt to provide an estimate of the incidence rates in heterosexuals, given the wide heterogeneity of this group. Specific surveys are needed to provide an estimate of heterosexuals at high risk of HIV infection among whom incidence rates may be very high, as suggested by a recent study conducted in the US [41]. It is interesting to note that the higher number of infections was estimated among heterosexuals while the number of diagnosed HIV infections classified as recent infections was higher among MSM. This is likely due to the different testing behaviour of these two groups, [42,43] and should be kept in mind when interpreting the results of observational studies that monitor the characteristics of recently acquired HIV infections. Furthermore, we should also consider that there is potential misclassification of MSM as heterosexual contact [44].

Although these results may be relevant to understanding recent trends of HIV epidemic in a low incidence country, several limitations should be considered.

The criteria used to define recent/non-recent infection are available only for persons diagnosed in SENDIH sites, and we assumed that incidence risk is the same for persons diagnosed in SENDIH and non-SENDIH sites. We assumed that individuals with missing information are missing at random and under this hypothesis the multiple imputation should have reduced potential bias in our estimates.

We combined three different criteria to define individuals with recent infection (i.e., an AI < 0.80, a previous documented negative test within 6 months, or laboratory evidence of recently acquired infection). In the model, we assumed that those identified as recent infection with the last two methods should have resulted with an AI < 0.80 if tested. Thus, it was reasonable to compute, also in this case, the incidence estimator as proposed by Karon. It is possible that some recent infections established with criteria other than AI, would have had an AI ≥ 08 and then classified as non-recent, but in this case we improved the sensitivity of the AI.

Other possible biases were described in detail in the article of Karon et al. [17] and these could also have biased our estimates. Among them, the AI test could have partly misclassified recent/non-recent infection because the accuracy of this test is not one, as assumed by the method. With regard to the reporting delays of HIV diagnoses, we can fairly exclude delays, since the period of interest was 2004-2008, the data were collected until the end of 2010 and the region considered is small.

The model assumes that the HIV test date is independent of the infection date for new testers, while for repeat testers it assumes that the risk of infection is constant between the last negative and first positive test date. Consequently the number of recent infections and thus incidence could be overestimated in people seeking HIV testing because of seroconversion symptoms or recent exposure. This issue could be addressed taking into account the reason for testing.

The history of testing and results of a TRI were not available for a significant proportion of new diagnoses. Preliminary analyses showed that results are strongly dependent on some parameters such as the window period of TRI used, the percentage of those with unknown testing history and the percentage of those without TRI result. We partly addressed this limit using multiple imputation that permits to correct the bias for missing at random information. Collecting information about HIV testing history and extending TRI to all new diagnoses reported to the regional surveillance system could improve the estimates limiting the effect of other source of bias.

Reliability of incidence estimates in specific subpopulations could be also affected by the accuracy of the subpopulation's size, such as IDU and MSM.

## Conclusion

In conclusion, this study combined surveillance data with TRI to provide an estimate of the incidence of HIV infections in recent years in an Italian region. These estimates suggest that the transmission of HIV infection in the region of Lazio remains frequent and interventions for prevention should be considered. In order to improve HIV incidence estimates, public health departments should initiate programs to increase the percentage of TRIs performed and other regions should incorporate TRI results into their surveillance systems.

## Abbreviations

AI: Avidity index; AIDS: Acquired immune deficiency syndrome; CI: Confidence interval; HIV: Human immunodeficiency virus; IDUM: Injecting drug user; MSM: Men who have sex with men; TRI: Test for recent infection.

## Competing interests

The authors declare that they have no competing interests.

## Authors' contributions

AM, PP, CA, NO, and EG conceived the initial idea and the study design; AM implemented the model and drafted the manuscript; PP and CA linked the data, contributed to data analysis and results interpretation; AN managed the database of SENDIH study and extracted the data; NO, VP, EG, and GI coordinated the SENDIH study and contributed to data interpretation; PP, AC, GG and PB coordinated the Regional Surveillance System and contributed to data interpretation; MRS and CS were responsible for the laboratory analyses. AM, PP, CA, NO and EG revised the manuscript. All authors read and approved the final manuscript.

## Pre-publication history

The pre-publication history for this paper can be accessed here:

http://www.biomedcentral.com/1471-2334/12/65/prepub
